# Proteinuria may be an indicator of adverse pregnancy outcomes in patients with preeclampsia: a retrospective study

**DOI:** 10.1186/s12958-021-00751-y

**Published:** 2021-05-14

**Authors:** Tingting Lei, Ting Qiu, Wanyu Liao, Kangjie Li, Xinyue Lai, Hongbo Huang, Rui Yuan, Ling Chen

**Affiliations:** 1 First Clinical College, Chongqing Medical University, Chongqing, China; 2 School of Public Health and Management, Chongqing Medical University, Chongqing, China; 3 Second Clinical College, Chongqing Medical University, Chongqing, China; 4 Chongqing Key Laboratory of Translational Research for Cancer Metastasis and Individualized Treatment, Chongqing Univ-ersity Cancer Hospital & Chongqing Cancer Institute & Chongqing Cancer Hospital, Chongqing, China; 5 The Center of Experimental Teaching Management, Chongqing Medical University, Chongqing, China

**Keywords:** Preeclampsia, Proteinuria, Pregnancy complications, Neonatal outcome, Gestational hypertension

## Abstract

**Background:**

Proteinuria is one of the common manifestations of patients with preeclampsia (PE), but whether the severity of proteinuria is related to the pregnancy outcome of patients with preeclampsia remains controversial. The present study aimed to determine the relationship between 24-h proteinuria and adverse outcomes in patients with preeclampsia.

**Methods:**

The present retrospective study included 329 pregnant women in Chongqing, China. Patients were divided into PE group and non-PE group. PE group was stratified into three subgroups based on the level of 24-h proteinuria. Correlation analysis was used to analyze the correlation between biochemical indexes and adverse pregnancy outcome, and Logistic regression analysis was used to analyze the risk factors of adverse pregnancy outcome. The receiver operating characteristic curve (ROC) was used to evaluate the ability of 24-h urinary protein to distinguish the adverse pregnancy outcome in patients with preeclampsia.

**Results:**

**(1)** Between PE and non-PE group, cesarean section rate in PE group was significantly higher than that in non-PE group (84.4% vs. 25.9%, *p* <  0.001). Laboratory findings such as uric acid and creatinine level in PE group were higher than those in non-PE group. **(2)** Among mild (proteinuria < 0.3 g/24 h), moderate (0.3 g/24 h ≦ proteinuria < 2 g/24 h) and massive (proteinuria ≧ 2 g/24 h) groups, the frequencies of induced labor (*p* = 0.006) and stillbirth (*p* = 0.002) increased with the increase of 24-h proteinuria. **(3)** Adverse outcomes were positively correlated with 24-h proteinuria (adverse maternal outcomes: *r* = 0.239, *p* = 0.002; adverse fetal outcomes: *r* = 0.336, *p* <  0.001). **(4)** The best 24-h proteinuria cutoff values to determine stillbirth, premature and fetal distress were 3965.0 mg/24 h, 984.75 mg/24 h and 1503.85 mg/24 h and their odds ratio (95% confidence interval) were 12.46 (3.46–44.88), 2.48 (1.15–5.37) and 10.02 (2.14–46.80), respectively.

**Conclusions:**

The severity of 24-h proteinuia may forecast adverse outcomes in women with preeclampsia. We suggest proteinuria should be retained as one of the monitoring indexes in patients with preeclampsia.

**Trial registration:**

Retrospectively registered. (LTMCMTS202001).

**Supplementary Information:**

The online version contains supplementary material available at 10.1186/s12958-021-00751-y.

## Background

Preeclampsia (PE), a kind of placental disease, is the most representative type of gestational hypertension. The incidence of preeclampsia accounts for 3–8% of all pregnancies, seriously affecting the health of mothers and babies [1]. Pregnant women with preeclampsia are prone to organ system dysfunction, leading to various adverse pregnancy outcomes, such as retinopathy, impaired renal function, preterm delivery, fetal growth restriction (FGR), etc. But the risk factors of adverse pregnancy outcomes need to be further explored [2–4]. A number of studies have shown that patients with preeclampsia should be wary of the occurrence of adverse pregnancy outcomes when massive proteinuria occurs [1, 3, 5]. In the past, the test of 24-h proteinuria was regarded as the gold standard for the diagnosis of preeclampsia in China [6]. However, proteinuria is no longer necessary for diagnosis according to guidelines issued by the American College of Obstetrics and Gynecology (ACOG) in 2013 and 2019 [7, 8]. In addition, many studies have suggested that the degree of proteinuria has nothing to do with the severity of preeclampsia, and there is no definite relationship between proteinuria and pregnancy outcome in patients with preeclampsia [9, 10]. It can be seen that the role of proteinuria in the diagnosis and evaluation of preeclampsia is still controversial. In this study, we retrospectively analyzed the relationship between 24-h proteinuria and adverse pregnancy outcome in patients with preeclampsia, and explored whether the degree of proteinuria affected the adverse pregnancy outcome in order to provide evidence for clinical diagnosis and treatment of preeclampsia.

## Methods

### Data sources

In present study, we retrospectively reviewed the clinical records of 329 pregnant women during January 2015 to July 2020 in Chongqing, China. There were 275 pregnant women diagnosed as preeclampsia and the other 54 pregnant women without preeclampsia were examined by obstetrics in the same period.

### Design

We stratified 329 pregnant women into PE group (pregnant women with preeclampsia, *n* = 275) and non-PE group (pregnant women without preeclampsia, *n* = 54). The clinical records of pregnant women at the time of admission and delivery were reviewed, including age, birth history, gestational weeks, mode of delivery, SBP, DBP, laboratory indexes (alanine aminotransferase, ALT; aspartate aminotransferase, AST; serum albumin, uric acid and creatinine.), adverse maternal outcomes, adverse fetal outcomes and other information.

#### PE group

(1) Inclusion criteria: ① All pregnant women meet the diagnostic criteria of preeclampsia. ② Singleton pregnancy. ③ There was no history of primary hypertension, nephropathy and diabetes. ④ The information was complete, including the detection of proteinuria.

(2) Exclusion criteria: ① Multiple pregnancies. ② Patients with primary hypertension, nephropathy and diabetes mellitus. ③ No delivery record due to spontaneous discharge or loss of follow-up. ④ Patients with incomplete laboratory and auxiliary examination data.

(3) Diagnostic criteria of preeclampsia: the diagnostic criteria of preeclampsia refers to the guidelines for diagnosis and treatment of hypertensive disorder in pregnancy (2015 Edition) [11]: Systolic blood pressure (SBP) ≧ 140 mmHg and (or) diastolic blood pressure (DBP) ≧ 90 mmHg after 20 weeks of gestation in a woman with a previously normal blood pressure, accompanied by any of the following: proteinuria ≧ 0.3 g per 24-h collection, or protein/creatinine ≧ 0.3, or dipstick reading ≧ 1+ (methods of examination when urine protein quantification is not possible), or in the absence of proteinuria, new-onset hypertension with the disorder in any one of the following organ or system: heart, lung, liver, kidney and other important organs, or abnormal changes in the blood system, digestive system, nervous system, placental fetal involvement and so on.

(4) Based on the 24-h proteinuria excretion, the PE group was divided into three subgroups: A: mild group, 24-h proteinuria < 0.3 g; B: moderate group, 24-h proteinuria 0.3 ~ 2 g; C: massive group, 24-h proteinuria ≧ 2 g.

#### Non-PE group

(1) Inclusion criteria: ① Patients with non-gestational hypertension and non-preeclampsia. ② Without proteinuria. ③ Singleton pregnancy. ④ The information was complete, including the detection of proteinuria.

(2) Exclusion criteria: ① Multiple pregnancy. ② Patients with primary hypertension, nephropathy and diabetes mellitus. ③ Those who have no delivery record due to spontaneous discharge or loss of follow-up. ④ The data of laboratory and auxiliary inspection are incomplete.

#### The definition of adverse pregnancy outcomes [10]

Adverse pregnancy outcomes are referred to severe complications threaten the life of pregnant women or fetus in patients with preeclampsia. Any of the following is defined as an adverse pregnancy outcome: (1) Adverse maternal outcome: Organ dysfunction (kidney, liver or heart failure), eclampsia, HELLP syndrome, hypoproteinemia, pleural or peritoneal effusion or pericardial effusion, oligohydramnios, placental abruption, maternal death, retinal disease, disseminated intravascular coagulation (DIC), postpartum hemorrhage. (2) Adverse fetal outcomes: fetal death, stillbirh, low birth weight, preterm delivery(<37 week), induced labor, fetal growth restriction (FGR), fetal distress, neonatal asphyxia, neonatal hypoxic encephalopathy, fetal malformation.

### Statistical analyses

The statistical analyses were performed by the statistical software SPSS24.0. Counting variables were expressed by frequency and analyzed by chi-square test; continuous variables were expressed by Xbar ± s and analyzed by *T*-test. Correlation analysis was used to analyze the correlation between biochemical indexes and adverse pregnancy outcome, and Logistic regression analysis was used to analyze the risk factors of adverse pregnancy outcome. The receiver operating characteristic curve (ROC) was used to evaluate the ability of 24-h proteinuria to distinguish the adverse pregnancy outcome in patients with preeclampsia. The difference was statistically significant (*P* <  0.05).

## Results

### Characteristics of included patients

Table 1 shows clinical characteristics of the 329 pregnant women. Among patients with PE, 170 patients measured 24-h proteinuria, and we divided them into 3 groups according to the degree of 24-h proteinuria (A vs. B vs. C). Group A was defined as proteinuria < 0.3 g/24 h (*n* = 53, mean 24-h proteinuria = 136.27 ± 89.39 mg/24 h), group B was defined as 0.3 to 2 g/24 h (*n* = 64, mean 24-h proteinuria = 884.29 ± 538.33 mg/24 h) and group C was defined as proteinuria excretion exceeding 2 g in 24 h (*n* = 53, mean 24-h proteinuria = 6246.13 ± 5099.54 mg/24 h).

**Table 1:** Characteristics of included women

	Non-PE	PE	*p* value	Subgroups^a^			*p* value
				A	B	C	
Number	54	275	–	53	64	53	–
**Clinical characteristics**							
Mean age (years)	27.61 ± 4.52	30.16 ± 5.53	< 0.001	31.70 ± 5.51	30.27 ± 6.00	31.28 ± 5.12	0.180
Gestation week (weeks)	37.96 ± 3.71	36.65 ± 3.68	0.012	37.19 ± 2.20	37.31 ± 2.87	34.53 ± 4.19^ab^	< 0.001
Primipara (%)	35 (64.8)	146 (53.1)	0.113	25 (47.2)	38(59.4)	27(50.9)	0.395
Multipara (%)	19 (35.2)	129 (46.9)		28 (52.8)	26 (40.6)	26 (49.1)	
**Mode of delivery**							
Spontaneously (%)	38 (70.4)	24 (8.7)	< 0.001	6 (13)	1 (1.7)	1 (2.4)	0.038
Caesarean section (%)	14 (29.6)	232 (91.3)		46 (87)	59 (98.3)	42 (97.6)	
**Laboratory findings**							
SBP (mmHg)	113.46 ± 9.67	146.34 ± 18.24	< 0.001	140.76 ± 14.23	143.67 ± 17.72	155.40 ± 19.53^ab^	< 0.001
DBP (mmHg)	70.5 ± 8.53	95.44 ± 13.68	< 0.001	91.30 ± 9.83	93.60 ± 13.10	104.15 ± 14.42^ab^	< 0.001
ALT (U/L)	14.58 ± 13.34	34.59 ± 64.46	< 0.001	37.79 ± 93.46	24.51 ± 25.83	43.62 ± 78.85	0.055
AST (U/L)	18.37 ± 6.89	35.82 ± 49.85	< 0.001	35.70 ± 67.53	30.01 ± 40.16	43.63 ± 64.72	0.191
Albumin (g/L)	37.44 ± 4.55	34.15 ± 4.92	< 0.001	36.63 ± 3.62	34.23 ± 4.38^a^	31.38 ± 4.52^ab^	< 0.001
Uric acid (μmol/L)	315.72 ± 67.35	424.38 ± 108.67	< 0.001	352.09 ± 82.85	419.66 ± 119.40^a^	460.14 ± 99.80^a^	< 0.001
Creatinine (μmol/L)	45.22 ± 6.23	62.53 ± 46.85	< 0.001	46.44 ± 8.46	58.53 ± 18.95	72.52 ± 41.09^ab^	< 0.001
Proteinuria (mg/24 h)	–	–	–	136.27 ± 89.39	884.29 ± 538.33	6246.13 ± 5099.54^ab^	< 0.001

*ALT* Alanine aminotransferasem, *AST* Aspartate aminotransferase, *DBP* Diastolic Blood Pressure; SBP: Systolic Blood Pressure; a: compared with A group, *p* <  0.05; b: compared with B group, *p* <  0.05

Subgroups^a^: A: 24-h urinary protein < 0.3 g; B:24-h urinary protein 0.3 ~ 2 g; C:24-h urinary protein ≧ 2 g

Between non-PE and PE groups, the maternal age and cesarean section rate were higher in PE group than those in non-PE group (*p <* 0.001). Laboratory findings such as SBP and DBP at admission, ALT, AST, uric acid and creatinine level in PE group were higher than those in non-PE group (*p* <  0.001), the albumin level was lower than that of non-PE group (*P* <  0.001).

Among three subgroups, C group had a shortest gestational week at delivery (*p <* 0.001). Laboratory findings such as SBP, DBP, uric acid and creatinine were found higher in C group than those in A and B group (*p* <  0.001). However, other clinical characteristics such as age, birth history, mode of delivery were similar in three groups.

### Comparison of adverse pregnancy outcomes in pregnant women

Table 2 shows the adverse maternal outcomes. The incidence of adverse maternal outcome in PE group (92/275, 33.5%) was more than that in non-PE group (2/54,3.7%), (*P* <  0.001). Compared with non-PE group, PE group had a higher incidence of hypoproteinemia (*p* = 0.006), organ dysfunction (*p* = 0.041), retinal disease (*p* = 0.047). Other adverse maternal outcomes such as pleural and peritoneal effusion, eclampsia, DIC, postpartum hemorrhage, placental abruption and maternal death had no significant difference between two groups (*p* > 0.05).

**Table 2:** Comparison of adverse maternal complications

	Non-PE	PE	*p* value	Subgroups^b^			*p* value
				A	B	C	
Number^a^	54	275		53	64	53	
**Mode of delivery**							
Spontaneously (%)	38 (70.4)	24 (8.7)	< 0.001	6 (11.3)	1 (1.7)	1 (5.7)	0.038
Caesarean section (%)	14 (25.9)	232 (84.4)		46 (86.8)	59 (92.2)	42 (79.2)	
Adverse maternal outcomes (%)	2 (3.7)	92 (33.5)	< 0.001	11 (21)	21 (32.8)	26 (49.1)	0.009
Retinal disease (%)	0	19 (6.9)	0.047	1 (1.9)	2 (3.1)	3 (5.7)	0.561
Hypoproteinemia (%)	1 (1.9)	44 (16)	0.006	3 (5.7)	8 (12.5)	19 (35.8)	< 0.001
Organ dysfunction^c^ (%)	0	20 (7.3)	0.041	3 (5.7)	0	7 (13.2)	0.010
Placental abruption (%)	0	10 (3.6)	0.155	1 (1.9)	3 (4.7)	1 (1.9)	0.578
Oligohydramnios (%)	0	29 (10.6)	0.012	6 (11.3)	8 (12.5)	3(11.3)	0.437
Effusion^d^ (%)	0	2 (0.7)	0.53	0	0	1 (1.9)	0.329
Eclampsia (%)	0	1 (0.4)	0.657	0	0	0	–
Postpartum hemorrhage (%)	2 (3.7)	3 (1)	0.151	0	1 (1.7)	0	0.435
DIC (%)	0	2 (0.7)	0.53	0	0	0	–
Hemorrhagic anemia (%)	1 (1.9)	6 (2.2)	0.878	0	0	1 (1.9)	0.329
Maternal death (%)	0	1 (0.4)	0.657	0	0	0	–

Number^a^: The number of multiple adverse pregnancy outcomes was counted according to the types of adverse pregnancy outcomes

Subgroups^b^: A: 24-h urinary protein < 0.3 g; B:24-h urinary protein 0.3 ~ 2 g; C:24-h urinary protein ≧ 2 g

Organ dysfunction^c^: renal dysfunction, hepatic dysfunction or cardiac insufficiency

Effusion^d^: including pleural or peritoneal effusion or pericardial effusion

In PE subgroups, the incidence of hypoproteinemia, organ dysfunction were significantly different among three groups (A: 5.7%; B: 12.5%; C: 35.8%; *p* <  0.001 for hypoproteinemia; A: 5.7%; B: 0%; C: 13.2%; *p* = 0.010 for organ dysfunction). No significant differences were observed in other maternal complications, such as placental abruption, retinal disease. No pregnant women died in the three groups.

### Comparison of adverse fetal outcomes among groups

Adverse fetal outcomes are shown in Table 3. The frequencies of adverse fetal outcomes were different in five groups (non-PE group: 24.1% vs. PE group: 45.1%, *p* = 0.004; group A: 30.2% vs. group B: 30.0% vs. group C: 67.9%, *p* <  0.001).

**Table 3:** Comparison of adverse fetal outcomes

	Non-PE	PE	*p* value	Subgroups^c^			*p* value
				A	B	C	
Number^a^	54	275	–	53	64	53	–
Adverse fetal outcomes (%)	13 (24.1)	124 (45.1)	0.004	16 (30.2)	19 (30.0)	36 (67.9)	< 0.001
Induced labour (%)	2 (3.7)	19 (6.9)	0.378	1 (1.9)	4 (6.2)	10 (18.9)	0.006
Premature (< 37 week)	3 (5.6)	56 (20.4)	0.010	15 (28.3)	7 (10.9)	18 (34.0)	0.009
FGR (%)	0	24 (8.7)	0.024	3 (5.7)	5 (7.8)	6 (11.3)	0.563
Low birth weight^b^ (%)	0	28 (10.2)	0.014	6 (11.3)	4 (6.2)	4 (7.5)	0.596
Oligohydramnios (%)	0	29 (10.5)	0.012	6 (11.3)	8 (12.5)	3 (5.7)	0.437
Stillbirth (%)	1 (1.9)	16 (5.8)	0.229	0	3 (4.7)	9 (17.0)	0.002
Neonatal asphyxia (%)	0	10 (3.6)	0.155	0	2 (3.1)	3 (5.7)	0.225
Neonatal hypoxic encephalopathy (%)	1 (1.9)	1 (0.4)	0.198	0	0	1 (1.9)	0.329
Fetal malformation (%)	2 (3.7)	2 (0.7)	0.068	1 (1.9)	0	1 (1.9)	0.043
Fetal distress (%)	4 (7.4)	23 (8.4)	0.815	1 (1.9)	4 (6.3)	5 (9.4)	0.253
Birth weight (g)	3157.46 ± 494.72	2878.16 ± 709.86	0.008	2872.02 ± 559.15	3010.25 ± 607.36	2444.76 ± 871.95	0.001

Number^a^: The number of multiple adverse pregnancy outcomes was counted according to the types of adverse pregnancy outcomes

Low birth weight^b^: including light for date infant and premature low birth weight infants, *FGR* Fetal Growth Restriction; Subgroups^c^: A: 24-h urinary protein < 0.3 g; B:24-h urinary protein 0.3 ~ 2 g; C:24-h urinary protein ≧ 2 g

Between non-PE and PE group, PE group had higher frequencies in premature (*p* = 0.010), FGA (*p* = 0.024), low birth weight (*p* = 0.014), and oligohydramnios (*p* = 0.012). Among another three subgroups, the rate of induced labour (*p* = 0.006), premature (*p* = 0.009), stillbirth (*p* = 0.001) and birth weight (*p* = 0.001) have significant difference. Though the incidence of these adverse outcomes didn’t increased with 24-h proteinuria degree, group C seemed to be more likely to develop into adverse fetal outcomes.

### Multivariate logistic regression analysis of related predictors and adverse pregnancy outcomes

In order to further determine the impact of proteinuria and other factors on pregnancy outcomes in patients with PE, a correlation test and binary logistics regression analysis were used to analyze parameters which got significance in the univariate analysis. Albumin, uric acid, creatinine, gestational weeks and proteinuria were associated with adverse maternal outcomes in PE patients (albumin: *r* = − 0.405, *p* <  0.001; uric acid: *r* = 0.304, *p* <  0.001; creatinine *r* = 0.235, *p* = 0.003; gestational weeks: *r* = − 0.236, *p* = 0.003; proteinuria: *r* = 0.239, *p* = 0.002). The factors highly related to the adverse fetal outcome were gestational weeks (*r* = − 0.463, *p* <  0.001) and proteinuria (*r* = 0.336, *p* <  0.001). Incorporate meaningful indicators of correlation analysis into multivariate logistic analysis, as shown in Table 4**,** the predictive indicators with statistically significant differences obtained by multivariate regression analysis were albumin, urine acid. Proteinuria was negatively correlated with gestational week (*r* = − 0.309, *p* <  0.001) and albumin (*r =* − 0.360, *p* <  0.001), suggesting that 24-h proteinuria may have an indirect predictive value for adverse outcomes.

**Table 4:** Multivariate logistic model predicting adverse outcomes of preeclampsia

	*B*	Standard deviation	WALD	Sig.	OR (95CI%)
**Adverse maternal outcomes**					
Constant	5.871	2.955	3.946	0.047	354.479
Albumin	−0.157	0.052	9.163	0.002	0.855 (0.773–0.946)
Urin acid	0.004	0.002	3.978	0.046	1.004 (1.000–1.009)
Proteinuria	0.000	0.000	0.178	0.673	1.000 (1.000–1.000)
Creatinine	−0.002	0.009	0.040	0.841	0.998 (0.980–1.016)
Gestational week	−0.079	0.056	1.956	0.162	0.924 (0.828–1.032)
**Adverse fetal outcomes**					
Constant	9.087	4.186	4.713	0.030	8842.119
Primipara	−0.163	0.452	0.129	0.719	0.850 (0.351–2.061)
Gestational week	−0.111	0.066	2.854	0.091	0.895 (0.787–1.018)
Proteinuria	0.000	0.000	0.395	0.530	1.000 (1.000–1.000)
SBP	0.013	0.019	0.513	0.474	1.013 (0.977–1.051)
DBP	−0.041	0.027	2.337	0.126	0.960 (0.911–1.012)
Albumin	−0.162	0.054	9.120	0.003	0.851 (0.766–0.945)
Urine acid	0.005	0.002	4.544	0.033	1.005 (1.000–1.009)
Creatinine	−0.002	0.010	0.041	0.839	0.998 (0.979–1.017)
ALT	−0.001	0.003	0.135	0.714	0.999 (0.992–1.006)

*DBP* Diastolic Blood Pressure, *SBP* Systolic Blood Pressure, *ALT* Alanine aminotransferase, *AST* Aspartate aminotransferase

### The best 24-h proteinuria cutoff in preeclampsia to determine adverse outcomes

**Fig. 1:**
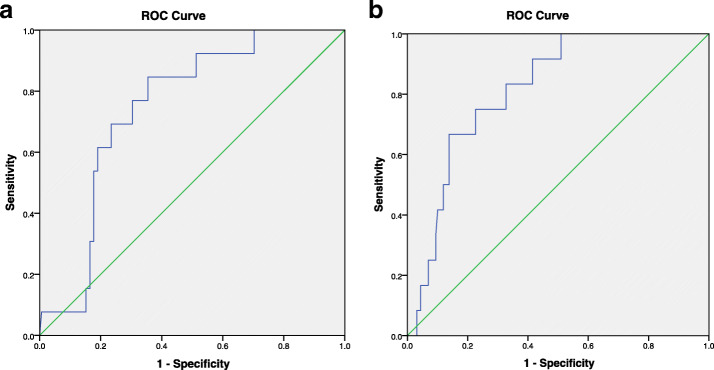
**a** 24-h proteinuria cutoff value to determine fetal distress. **b** 24-h proteinuria cutoff value to determine stillbirth

While studying the relationship between 24-h proteinuria and adverse outcomes in women with preeclampsia, we found there were differences in the incidence of hypoalbuminemia, organ dysfunction, premature delivery and stillbirth in different level of 24-h proteinuria, so we explore the potential diagnostic value of 24-h proteinuria to predict adverse outcomes. Figure 1 (1a to 1b) showed the best 24-h proteinuria cutoff based on ROC curves. The cutoff values to determine stillbirth, premature and fetal distress were 3965.0 mg/24 h, 984.75 mg/24 h and 1503.85 mg/24 h, those corresponding area under the ROC curve (AUC) were 0.816, 0.618 and 0.745 (*p* <  0.001, *p* = 0.034 and *p* = 0.003, respectively) and odds ratios (ORs) (95% confidence interval, CIs) were 12.46 (3.46–44.88), 2.48 (1.15–5.37) and 10.02 (2.14–46.80), respectively. Although univariate analysis showed that there was a difference in the incidence of organ dysfunction among three subgroups, the cutoff value obtained by ROC curve was not significant.

## Discussion

Previous studies have shown that the degree of 24-h proteinuria is positively correlated with the severity of PE and the occurrence of adverse pregnancy outcomes [5, 12, 13]. Therefore, proteinuria was once considered to be one of the necessary indicators for clinical diagnosis of PE [6]. However, in recent years, the necessity of proteinuria in the diagnosis of PE has been controversial. In a 2010 study [14], researchers showed that PE patients with proteinuria had higher blood pressure and cesarean section rates than PE patients without proteinuria, but proteinuria was not a predictor of adverse pregnancy outcomes. The ACOG (2013) [7] adjusted proteinuria from a necessary index to an additional index in the diagnosis of PE, and deleted 24-h proteinuria ≥5 g from the diagnostic criteria of severe PE since only a minimal relationship existed between proteinuria and adverse pregnancy outcomes. In the latest guideline ACOG (2019) [8], this change in diagnostic criteria is still retained. It suggests that although preeclampsia is usually accompanied by new-onset proteinuria, hypertension and other signs or symptoms of preeclampsia may present in some women without proteinuria. In the absence of proteinuria, new-onset hypertension with the new onset of any of the following can be diagnosed as preeclampsia: thrombocytopenia, renal insufficiency, impaired liver function, pulmonary edema. Chinese guideline for diagnosis and treatment of hypertensive disorder in pregnancy (2015 Edition) also pointed out that proteinuria was not a decisive factor in the diagnosis of PE, but played an important role in evaluating the prognosis of PE [11]. In 2018, The International Society for the Study of Hypertension in Pregnancy (ISSHP) defined presence of proteinuria is not necessary to diagnose preeclampsia [15]. At the same time, some scientists still hold the opinion that proteinuria is closely related to adverse pregnancy outcome in patients with preeclampsia, but few studies proved the relationship between the degree of proteinuria and a specific perinatal / neonatal outcome in women with preeclampsia.

In present study, the rate of cesarean section in PE group was significantly higher than that in non-PE group. This could be because some patients with preeclampsia need to terminate the pregnancy early, but the cervix is not mature, or in severe cases, the pregnancy needs to be terminated immediately. Besides, our results showed the incidence of adverse outcome in PE group was higher than that in non-PE group, including hypoalbuminemia, oligohydramnios, organ dysfunction, retinal disease, premature delivery, FGR, low birth weight, this result is consistent with Homer’s [16], suggesting that PE could be one of the factors affecting pregnancy outcome. PE is a systemic vascular disorder characterized by new-onset hypertension and proteinuria after 20 weeks of gestation, and its basic pathophysiological lesion is systemic arteriolar spasm. Therefor, this disease can affect almost every organ system and lead to complications related to preeclampsia [17]. When renal arterioles are affected, patients may develop a decrease of renal blood perfusion and glomerular filtration rate, as the damage progresses, the basement membrane cells and vascular endothelial cells can be damaged, which results in increased vascular permeability, massive protein loss and proteinuria [17]. A large amount of proteinuria makes pregnant women prone to hypoproteinemia and organ dysfunction. Retinal arteriole spasm can easily lead to retinal detachment. In addition, pregnant women are in a state of hypercoagulability during pregnancy, and the vascular endothelial cell injury in patients with preeclampsia further leads to microvascular thrombosis. Oligohydramnios in patients with preeclampsia may be caused by placental microthrombosis [18]. Combined with the results of the study and the pathological features of PE, proteinuria may be a potential index to predict pregnancy outcomes in preeclampsia patients.

In this study, non-PE patients were limited to undiagnosed preeclampsia and without proteinuria, while not all PE patients had proteinuria according to the 2015 diagnostic guidelines, and there were differences in basic conditions and a variety of biochemical indexes between the two groups, which could not prove a clear relationship between adverse outcome and proteinuria of PE patients. Considering this potential limitation, we performed subgroup analysis and correlation analysis on patients with PE who underwent 24-h proteinuria detection to further explore the relationship between urinary protein and pregnancy outcome. We analyzed 175 PE patients with proteinuria in diffrent degree, and found differences exsisted in the incidence of induced labor, hypoalbuminemia, organ dysfunction, premature delivery and stillbirth in pregnant women with 24-h proteinuria. Contrary to Newman’s [19], Sérgio Hofmeister Martins-Costa’s [20] findings, their studies showed that excessive proteinuria did not mean a more serious outcome, and the increased proteinuria had nothing to do with the risk of adverse outcomes, but in present study, we found the increase of proteinuria could add the rate of adverse outcome of preeclampsia. Moreover, with the increase of the degree of 24-h proteinuria, the incidence of induced labor and stillbirth increased. These results demonstrate that proteinuria may be one of the factors of adverse pregnancy outcome in patients with preeclampsia and this result is consistent with Kumari’s [21] and Chan’s [22].

As reported in our results, the massive group had a shorter gestational week and a higher premature birth rate, but it is still unknown whether this condition is the result of doctors’ choice or whether it is caused by the disease process itself, so we pay more attention to the adverse outcomes that are less affected by artificial manipulation. We used correlation analysis to explore the relative factors of adverse pregnancy outcome in patients with preeclampsia. The results showed that 24-h proteinuria, serum albumin, creatinine, uric acid and gestational week were all related to adverse pregnancy outcome. Binary logistic regression analysis results showed the predictive indicators with statistically significant differences were albumin, urine acid. The accuracy of the model predicting the adverse maternal outcome and adverse fetal outcome were 72.6, 68.2%, respectively. In our study, 24-h proteinuria was negatively correlated with albumin, it means we cannot deny the role of 24-h proteinuria in predicting adverse outcomes in patients with preeclampsia.

For decades, few studies have demonstrated a clear relationship between the degree of proteinuria and a specific perinatal / neonatal outcome in women with preeclampsia. In 2020, Mamoru Morikawa et al. [23] first demonstrated the relationship between the severity of the Protein: Creatinine (P/C) ratio at delivery and perinatal / neonatal outcomes among women with preeclampsia based on a ROC curve, the best P/C ratio cutoff values in preeclampsia to determine early preterm birth (EPB) and maternal central serous chorioretinopathy (CSC) were 4.1 and 4.8, respectively. The results indicated that the best cutoff value of the P/C ratio at delivery for worsened perinatal outcomes in women with preeclampsia was 4.8. In present study, we used the ROC curve to analyze the relationship between 24-h proteinuria and adverse outcomes of patients with preeclampsia, and obtained the corresponding cutoff value. The cutoff value to determine hypoproteinemia, stillbirth, preterm birth and intrauterine distress were 1935 mg/24 h, 3965.0 mg/24 h, 984.75 mg/24 h and 1503.85 mg/24 h, respectively. Obviously, the cutoff values of 24-h proteinuria varies from different adverse outcomes, this may be one of the reasons for the controversy in the application of urine protein in preeclampsia. These cutoff values may be limited in clinical application, but it is undeniable that proteinuria has been once again confirmed to be associated with adverse outcomes, which also suggests that doctors still need to be vigilant about proteinuria in the treatment of patients with PE.

## Conclusions

In conclusion, the increase of 24-h proteinuria may lead to an increase in the incidence of adverse outcomes in women with preeclampsia. We suggest that proteinuria should be retained as one of the monitoring indexes for patients with preeclampsia. However, the pregnancy outcome of patients with preeclampsia is affected by many factors, so the monitoring of proteinuria combined with the consideration of complications and other laboratory indicators may have a more accurate prediction of adverse pregnancy outcomes.

## Supplementary Information

**Additional file 1: **Correlation test between various diagnostic indicators and adverse outcomes. Relationship between 24 h urine protein cutoff value based on ROC curve and pregnant outcomes. **Fig1.** 24 h proteinuria cutoff value to determing hypoproteinemia. **Fig2.** 24 h proteinuria cutoff value to determine adverse outcomes.

## Data Availability

Not applicable.

## Abbreviations

ACOG: American College of Obstetricians and Gynecologists
ALT: Alanine Aminotransferase
AST: Aspartate aminotransferase
AUC: Area Under Curve
CI: Confidence Interval
DBP: Diastolic Blood Pressure
DIC: Disseminated Intravascular Coagulation
EPB: Early Preterm Birth
FGR: Fetal Growth Restriction
ORs: Odds Ratio
P/C Ratio: Protein Creatinine Ratio
PE: Pre-eclampsia
ROC: The Receiver Operating Characteristic curve
SBP: Systolic Blood Pressure
