# Supplementary material for: Proteinuria may be an indicator of adverse pregnancy outcomes in patients with preeclampsia: a retrospective study
Source: Reprod Biol Endocrinol. 2021 May 14;19:71. doi: 10.1186/s12958-021-00751-y (PMC8120921; doi:10.1186/s12958-021-00751-y)
Supplement: Supplementary file 1 — Additional file 1: Correlation test between various diagnostic indicators and adverse outcomes. Relationship between 24 h urine protein cutoff value based on ROC curve and pregnant outcomes. Fig1. 24 h proteinuria cutoff value to determing hypoproteinemia. Fig2. 24 h proteinuria cutoff value to determine adverse outcomes. [file 12958_2021_751_MOESM1_ESM.docx]

**Correlation test between various diagnostic indicators and adverse outcomes**

|  |  | Gestational weeks | 24-hour urine protein (mg/24h) | SBP | DBP | Albumin | Urine acid | Creatinine |
| --- | --- | --- | --- | --- | --- | --- | --- | --- |
| *r* | Maternal complications | -0.236 | 0.239 | 0.057 | 0.026 | -0.405 | 0.304 | 0.235 |
| *p* |  | 0.003 | 0.002 | 0.256 | 0.381 | <0.001 | <0.001 | 0.003 |
| *r* | Fetal complications | -0.463 | 0.345 | 0.244 | 0.328 | -0.296 | 0.228 | 0.145 |
| *p* |  | <0.001 | <0.001 | 0.002 | <0.001 | <0.001 | 0.004 | 0.048 |
| *r* | 24h proteinuria | -0.309 | 1 | 0.226 | 0.290 | -0.360 | 0.197 | 0.166 |
| *p* |  | <0.001 | - | 0.004 | <0.001 | <0.001 | 0.020 | 0.052 |

**Relationship between 24hour urine protein cutoff value based on ROC curve and pregnant outcomes.**

|  | | 24hour urine protein cutoff value（mg/24h） | Se | Sp | OR | 95% CI of  OR | Youden  index | AUC | Standa-rd error | *P* | 95%CI |
| --- | --- | --- | --- | --- | --- | --- | --- | --- | --- | --- | --- |
| Adverse outcomes | 872.09 | 0.63 | 0.76 | 5.03 | 2.60-9.74 | 0.386 | 0.698 | 0.041 | <0.001 | 0.618-0.777 |  |
| Hypoproteinemia | 1935 | 0.72 | 0.74 | 6.7 | 2.6-17.4 | 0.46 | 0.740 | 0.057 | <0.001 | 0.629-0.851 |  |
| Stillbirth | 3965.0 | 0.67 | 0.86 | 12.46 | 3.46-44.88 | 0.529 | 0.816 | 0.048 | <0.001 | 0.722-0.910 |  |
| Premature | 984.75 | 0.62 | 0.60 | 2.48 | 1.15-5.37 | 0.224 | 0.618 | 0.054 | 0.034 | 0.511-0.724 |  |
| Intrauterine distress | 1503.85 | 0.85 | 0.66 | 10.02 | 2.14-46.80 | 0.492 | 0.745 | 0.055 | 0.003 | 0.638-0.853 |  |

Se, Sensitivity; Sp, Speciality; OR, Odds Ratio; CI: Confidence Interval; AUC: Area Under Curve.


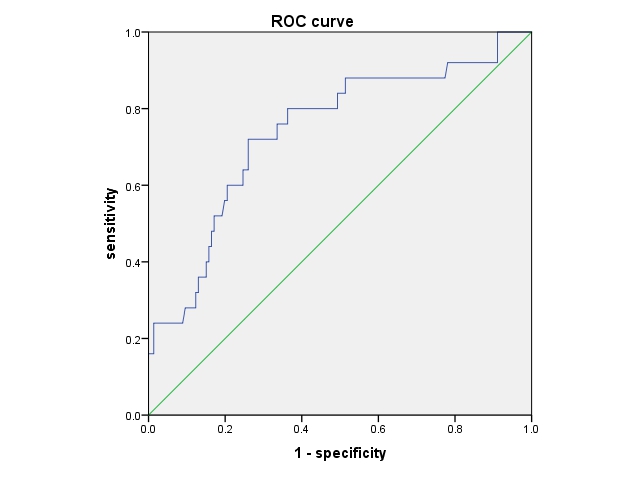


Fig1. 24h proteinuria - Hypoproteinemia.
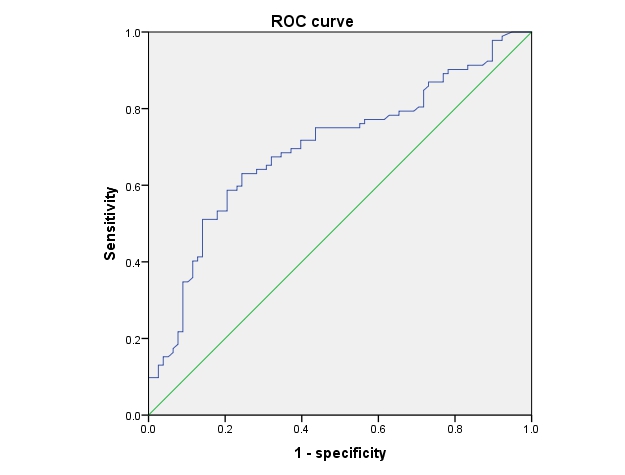


Fig2. 24h proteinuria - adverse outcomes
